# Assessment of Peak Oxygen Uptake with a Smartwatch and its Usefulness for Training of Runners

**DOI:** 10.1055/a-1686-9068

**Published:** 2022-01-30

**Authors:** Peter Düking, Bas Van Hooren, Billy Sperlich

**Affiliations:** 1 Integrative and Experimental Exercise Science, Department of Sport Science, University of Würzburg, Würzburg, Germany; 2 Department of Nutrition and Movement Sciences, NUTRIM School of Nutrition and Translational Research in Metabolism, Maastricht University Medical Centre+, Maastricht, Netherlands

**Keywords:** data-guided training, digital health, digital training, eHealth, innovation, technology, wearable, mHealth

## Abstract

Peak oxygen uptake (˙VO _2peak_ ) is an important factor contributing to running performance. Wearable technology may allow the assessment of ˙VO _2peak_ more frequently and on a larger scale. We aim to i) validate the ˙VO _2peak_ assessed by a smartwatch (Garmin Forerunner 245), and ii) discuss how this parameter may assist to evaluate and guide training procedures. A total of 23 runners (12 female, 11 male; ˙VO _2peak_ : 48.6±6.8 ml∙min ^−1^ ∙kg ^−1^ ) visited the laboratory twice to determine their ˙VO _2peak_ during a treadmill ramp test. Between laboratory visits, participants wore a smartwatch and performed three outdoor runs to obtain ˙VO _2peak_ values provided by the smartwatch. The ˙VO _2peak_ obtained by the criterion measure ranged from 38 to 61 ml∙min ^−1^ ∙kg ^−1^ . The mean absolute percentage error (MAPE) between the smartwatch and the criterion ˙VO _2peak_ was 5.7%. The criterion measure revealed a coefficient of variation of 4.0% over the VO2peak range from 38–61 ml∙min ^−1^ ∙kg ^−1^ . MAPE between the smartwatch and criterion measure was 7.1, 4.1 and −6.2% when analyzing ˙VO _2peak_ ranging from 39–45 ml∙min ^−1^ ∙kg ^−1^ , 45–55 ml∙min ^−1^ ∙kg ^−1^ or 55–61 ml∙min ^−1^ ∙kg ^−1^ , respectively.

## Introduction

Peak oxygen uptake (˙VO _2peak_ ) is extensively investigated among individuals of different age, gender, and performance levels 1 2 3 4 and is a key component of endurance performance in heterogeneous populations. Although ˙VO _2peak_ does not predict performance in homogeneous groups of athletes (i. e., elite level) and while changes in ˙VO _2peak_ allows predicting some but not all changes in endurance performance 5 , an exceptionally high ˙VO _2peak_ constitutes a prerequisite for competitive success in endurance athletes 3 6 . Based on the peak values, percentages of ˙VO _2peak_ are often applied in sports practice to prescribe training intensity, although they are subject to current scientific debate 7 .

Maintaining or improving ˙VO _2peak_ is an important goal in the training process of runners. Since individuals show considerable inter- and intra-individual physiological responses to the same training procedures 2 8 , frequent evaluation of the effectiveness of training procedures and responsive adjustments of training procedures are required by evaluating important performance indicators (such as ˙VO _2peak_ and others).

The accurate assessment of ˙VO _2peak_ requires i) time-consuming and expensive laboratory setup for gas exchange measurement, ii) specialized laboratory staff, and iii) an all-out effort by the participant. These disadvantages impair frequent assessment of ˙VO _2peak_ , especially for recreational runners without access to such equipment. These limitations might be surpassed by advancements in the field of wearable sensors (e. g., smartwatches) and accompanying machine learning algorithms intended to assess ˙VO _2peak_ . Wearable sensors used in research settings (e. g., a combination of an accelerometer worn on the tibia and a heart rate sensor) employing a mixed-effects unpenalized linear regression model allow the estimation of ˙VO _2peak_ with an error of 4.92% in the laboratory 9 . Nevertheless, these sensors and algorithms may not be available to the public, and few studies have evaluated the validity of ˙VO _2peak_ measurements with end consumer wearables (e. g., smartwatches) 10 11 . However, frequent hard- and software developments of end consumer devices likely affect data quality, and therefore it is important to regularly evaluate these devices for daily application 12 13 . Regarding the daily use of this technology and data, another challenge is to interpret and draw physiologically meaningful conclusions for training procedures. In this regard, recreational runners will need some level of knowledge on how to interpret changes in ˙VO _2peak_ to guide their training 14 .

The goal of the present investigation is twofold: i) to validate the ˙VO _2peak_ provided by an end consumer smartwatch (Garmin Forerunner 245) against a common criterion measure, and ii) to briefly discuss the usefulness and shortcomings of ˙VO _2peak_ measurements to guide a runner’s training.

## Materials and Methods

### Participants

Twenty-three non-competitive recreational runners (11 men, 12 women, mean age 23±3 years, body height 173±8 cm, body mass 70.1±11.2 kg; ˙VO _2peak_ : 48.6±6.8 ml/min/kg; training characteristics: 2–3 times per week for 45 min at a self-perceived low intensity) of Caucasian origin were informed about all experimental procedures and provided written consent to participate. The study was approved by the institute’s ethical committee and performed in accordance with the declaration of Helsinki and the study follows ethical standards in sport and exercise science research 15 .

### Experimental procedures

**Fig. 1:**
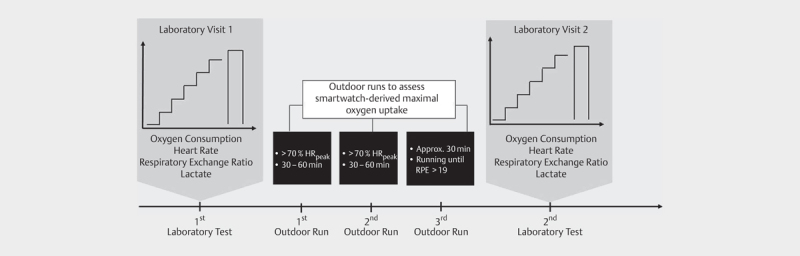
Experimental procedure. Laboratory visit: Ramp protocol to assess maximal oxygen uptake by the criterion. Initial speed set to 7 km∙h ^−1^ , increasing every minute by 1 km∙h ^−1^ . Outdoor runs to assess smartwatch derived maximal uptake.

The experimental procedure is illustrated in Fig. 1 .

All participants reported twice (7–10 days apart) to the laboratory for assessment of anthropometric data, maximal heart rate, and ˙VO _2peak_ . Even with gold-standard criterion measures, there is an error stemming from technical error and random within-subject variation 16 . To assess the error of the criterion measure in our sample, we tested each participant twice with the criterion measure in the laboratory. This repeated measure allows calculating i) the mean ˙VO _2peak_ values of both laboratory visits, which delivers a better estimation of an individual’s ˙VO _2peak_ ; and ii) the reliability of the gold-standard criterion-measures allowing comparison to the validity error between the criterion and the smartwatche-derived ˙VO _2peak_ .

To assess ˙VO _2peak_ provided by the smartwatch, the manufacturer’s instructions for use indicate a person should run outdoors for at least 10 min with a heart rate “several minutes” above 70% of the maximal heart rate 17 . The manufacturer indicates that the ˙VO _2peak_ assessment might improve following “a couple” of runs 17 . Therefore, between both laboratory visits, all runners performed three outdoor runs (longer than 30 min) on flat terrain.

### Ramp test protocol for assessment of peak oxygen uptake

Each participant performed a ramp protocol on a motorized treadmill (Mercury, h/p/cosmos sports and Medical GmbH, Nussdorf-Traunstein, Germany) to assess ˙VO _2peak_ . Initially the treadmill speed was set to 7 km∙h ^−1^ increasing every minute by 1 km∙h ^−1^ until volitional exhaustion. In our experience, this ramp slope (i. e., km∙h ^−1^ increment) allows recreational runners to reach exhaustion within approximately 10–15 min, which is important for accurate assessment of ˙VO _2peak_ 7 . Exhaustion was verified if three of the four following criteria were met: 1) plateau in ˙VO _2_ , that is, an increase<1.0 mL∙min ^−1^ ∙kg ^−1^ despite an increase in velocity; 2) respiratory exchange ratio>1.1; 3) rating of perceived exertion>18; and 4) peak blood lactate (peak lactate)>6 mmol∙L ^−1^ 30 s after ramp testing. After completion of the ramp test, the participants performed passive recovery for 5 min followed by an instantaneous step increase in running velocity (verification phase) corresponding to 105% of the velocity achieved during the ramp test. The verification phase ended with each runner’s individual volitional exhaustion 18 . The ˙VO _2peak_ values, assessed by averaging the last 30 s of the ramp and verification run, were compared 18 and the higher value was used for further analysis.

### Assessment of smartwatch derived peak oxygen uptake

Each runner wore two smartwatches, one the left wrist and one on the right. This allowed us to obtain estimates for ˙VO _2peak_ from two independent smartwatches at the same time. All participants were instructed to perform three outdoor runs at a constant pace without stopping. To align with the manufacturers recommendations and to ensure that each participant ran “several minutes” above 70% of peak heart rate (for the first two runs), they all were instructed to run for 30–60 min until exhaustion (i. e.,>18 on the Borg scale). For the third run, the runners were instructed to run for 30 min until fully exerted. We assessed the level of exhaustion by the rating of perceived exertion (RPE) 19 , which all runners reported approximately 20 min after completing the running session.

### Criterion measure

A portable breath-by-breath analyzer (Metamax 3B, CORTEX Biophysik GmbH, Leipzig, Germany) served as the criterion measure. The oxygen sensor of this portable breath-by-breath gas analyzer provides reliable data with technical measurement error below 2% 20 .

### Smartwatch

An end consumer smartwatch (Forerunner 245, Garmin, Olathe, USA) employing an optical heart rate sensor as well as a GPS receiver unit was used for this study. We chose the optical heart rate sensor (and not an electrical chest belt sensor) as the optical sensors are becoming more readily available and when optical sensors prove scientific trustworthiness, it is likely that runners will choose this type of sensor due to greater comfort compared to a chest strap. The smartwatch was programmed as indicated by the manufacturer. We did not enter the participants’ maximum heart rate into the software since many recreational runners do not know their actual individual maximum heart rate. The exact algorithms of ˙VO _2peak_ assessment are not disclosed by the manufacturer, yet it is indicated that reliable heart and GPS-derived velocity data segments from individual runs are used to estimate ˙VO _2peak_ 21_._

### Statistical analysis

A dependent *t* -test (performed in the Statistica Software package for Windows Version 7.1) assessed the difference in peak oxygen uptake between the two exercise tests. An alpha level of≤0.05 was considered statistically significant.

### Reliability of the criterion measure

As previously performed 22 , reliability of the criterion measure ˙VO _2peak_ was calculated as the percentage change in the mean (CM%) and typical error (TE%) expressed as a coefficient of variation (CV%), calculated as SD of the percentage change scores between repeated measures divided by the square root of 2. The intraclass correlation coefficient (ICC, 3.1) was calculated and interpreted according to 23 in order to examine overall group-level association. ICC values less than 0.5, between 0.5 and 0.75, between 0.75 and 0.9, and greater than 0.90 are indicative of poor, moderate, good, and excellent reliability, respectively 23 . For all measures, the corresponding 95% CI were calculated.

### Validity analysis comparing the end consumer smartwatch against the criterion measure

To investigate the validity of the ˙VO _2peak_ provided by the smartwatch, we averaged the ˙VO _2peak_ of the three runs. We also split the sample in runners with low (˙VO _2peak_ ≤45 ml∙kg ^−1^ ∙min ^−1^ ), medium (˙VO _2peak_ 45–55 45 ml∙kg ^−1^ ∙min ^−1^ ), and high (˙VO _2peak_ ≥55 45 ml∙kg ^−1^ ∙min ^−1^ ) ˙VO _2peak_ to evaluate whether the validity differed between the subgroups. As no international standards exist for thresholds of low, medium, and high ˙VO _2peak_ categories these levels are arbitrary. To additionally examine the validity of several runs, we calculated all statistical parameters mentioned in this section for ˙VO _2peak_ values that were given for each of the three outdoor runs.

As previously performed, mean absolute percent errors (MAPE) were calculated to provide an indicator of overall measurement error 24 . MAPE was calculated as the average of absolute difference between the smartwatch and the criterion measure divided by the criterion measure value, multiplied by 100.

Bland–Altman plots display the corresponding 95% limits of agreement and fitted lines (from regression analyses between mean and difference) with their corresponding parameters (i. e., intercept and slope). A fitted line that provides a slope of 0 and an intercept of 0 exemplifies perfect agreement 24 .

## Results

All descriptive statistics of the laboratory tests and the outdoor runs are summarized in Tables 1 and 2 .

**Table:** **Table 1** Descriptive statistics of the main variables obtained during the 1 ^st^ and 2 ^nd^ laboratory tests (mean±SD).

	1 ^st^ Laboratory test	2 ^nd^ Laboratory test	Average of laboratory tests
Peak oxygen uptake [ml∙min ^−1^ ∙kg ^−1^ ]	47.5±6.8	49.7±7.3	48.6±7.0
Peak heart rate [bpm]	196±9	193±8	195±8
Peak respiratory exchange ratio	1.14±0.06	1.13±0.26	1.14±0.18
Peak blood lactate concentration [mmol∙L ^−1^ ]	7.0±1.9	6.1±1.5	6.6±1.9
Completed stages on treadmill [n]	7.7±2.4	7.8±2.3	7.5±2.3

**Table:** **Table 2** Descriptive statistics of variables obtained during the outdoor runs (mean±SD).

	1 ^st^ Run	2 ^nd^ Run	3 ^rd^ Run	Average of all runs
Duration [s]	2437±608	2456±676	1784±59	2232±614
Distance [km]	6.99±2.17	7.08±2.41	5.89±0.93	6.67±2.01
Mean velocity [m∙s ^−1^ ]	2.86±0.41	2.86±0.60	3.30±0.96	3.00±0.71
Mean heart rate [bpm]	161.3±27.5	161.3±28.8	172.5±36.6	164.9±31.4
Mean heart rate [% of peak heart rate]	82.7%	82.7%	88.4%	84.5%
Mean ratings of perceived exertion [Borg 6–20 scale]	16±2	16±2	18±2	16±2

The mean ˙VO _2peak_ of the two criterion tests were 47.5 ±6.8 ml∙min ^−1^ ∙kg ^−1^ and 49.7±7.2 ml∙min ^−1^ ∙kg ^−1^ (average of laboratory assessed ˙VO _2peak_ : 48.6±7.0 ml∙min ^−1^ ∙kg ^−1^ ) and this difference was significant (p=0.0003) between the two tests.

We had to discard three ˙VO _2peak_ estimations derived from the smartwatches due to handling errors occurring with the smartwatch. The mean ˙VO _2peak_ estimated by the smartwatch after the first, second, and third run as well as the mean of all runs was 49.1 ±4.6 ml∙min ^−1^ ∙kg ^−1^ , 49.0±4.7 ml∙min ^−1^ ∙kg ^−1^ , 48.3±9.0 ml∙min ^−1^ ∙kg ^−1^ , 49.1±4.6 ml∙min ^−1^ ∙kg ^−1^ , respectively.

Overall, the criterion measure showed a CM% of 4.6 (95%CI −3.1 to 7.4), a TE% as CV% of 4.0 (95%CI −0.7 to 4.7). The TE% as CV% of 4.0 corresponds to an error of 1.96 ml∙min ^−1^ ∙kg ^−1^ . The ICC of 0.943 (95%CI 0.736 to 0.982) indicates excellent reliability. When splitting the ˙VO _2peak_ into subgroups of lower (˙VO _2peak_ ≤45 ml∙min ^−1^ ∙kg ^−1^ ; n=12), medium (˙VO _2peak_ 45–55 ml∙min ^−1^ ∙kg ^−1^ ; n=13), and higher (˙VO _2peak_ ≥55 ml∙min ^−1^ ∙kg ^−1^ ; n=13) ˙VO _2peak_ , the criterion measure showed a TE% as CV% of 2.6% (95%CI −0.1 to 2.7); 3.5 (95%CI 0.0 to 3.8) and 4.0% (95%CI 0.8 to 6.3).

When averaging the ˙VO _2peak_ values of all three runs, the smartwatch showed a MAPE of 5.7% (corresponding to an error of 2.80 ml∙min ^−1^ ∙kg ^−1^ ). When the ˙VO _2peak_ provided by the smartwatch following the first, second, and third outdoor run were compared, the MAPE was 5.7% (corresponding to an error of 2.80 ml∙min ^−1^ ∙kg ^−1^ ), 5.6% (corresponding to an error of 2.70 ml∙min ^−1^ ∙kg ^−1^ ) and 5.6% (corresponding to an error of 2.70 ml∙min ^−1^ ∙kg ^−1^ ).

**Fig. 2:**
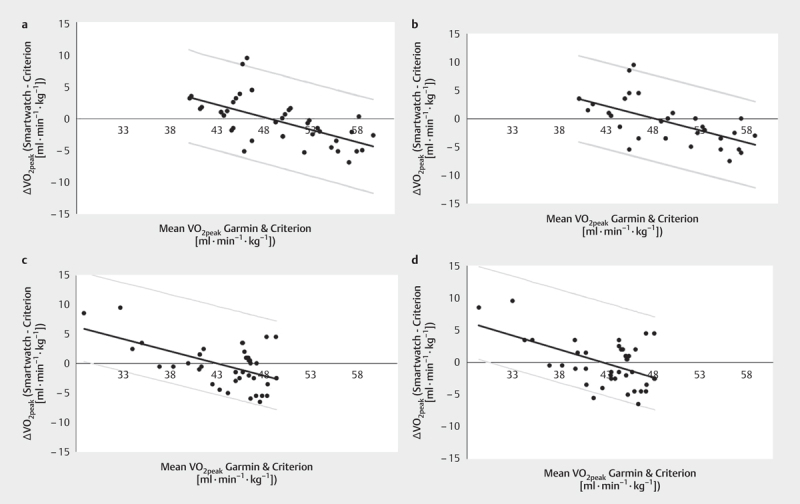
Bland-Altman plots (mean ˙VO _2peak_ of the criterion vs. mean ˙VO _2peak_ of the smartwatch) for **a** mean values of 3 outdoor runs, **b** only the first run, **c** only the second run, **d** only the third run.

The Bland-Altman plot is displayed in Fig. 2 .

When the ˙VO _2peak_ were split into subgroups of lower (˙VO _2peak_ ≤45 ml∙min ^−1^ ∙kg ^−1^ ), medium (˙VO _2peak_ 45–55 ml∙min ^−1^ ∙kg ^−1^ ), and higher (˙VO _2peak_ ≥55 ml∙min ^−1^ ∙kg ^−1^ ) ˙VO _2peak_ , the smartwatch showed a MAPE of 7.1% (corresponds to an error of 3.48 ml∙min ^−1^ ∙kg ^−1^ ), 4.1% (corresponds to an error of 2.01 ml∙min ^−1^ ∙kg ^−1^ ) and −6.2% (corresponds to an error of −3.04 ml∙min ^−1^ ∙kg ^−1^ ), respectively.

## Discussion

The primary goal of the present investigation was to validate the ˙VO _2peak_ provided by an end consumer smartwatch (Garmin Forerunner 245) against a common criterion measure. The two main findings are:

1. Over the ˙VO _2peak_ range of 38 to 61 ml∙min ^−1^ ∙kg ^−1^ (as measured by the criterion measure), the overall MAPE between the smartwatch and the criterion is 5.7% (~2.8 ml∙min ^−1^ ∙kg ^−1^ ). The MAPE does not seem to decrease when performing one, two or three runs.
2. When clustering the runners’ ˙VO _2peak_ (i. e., 39 to 45 ml∙min ^−1^ ∙kg ^−1^ , 45 to 55 ml∙min ^−1^ ∙kg ^−1^ , and 55 to 61 ml∙min ^−1^ ∙kg ^−1^ ) the MAPE is 7.1% (~ 3.5 ml∙min ^−1^ ∙kg ^−1^ ), 4.1% (~ 2.0 ml∙min ^−1^ ∙kg ^−1^ ) and −6.2% (~ −3.0 ml∙min ^−1^ ∙kg ^−1^ ), indicating that within the lower ˙VO _2peak_ category, the smartwatch tends to overestimate the runners’ actual ˙VO _2peak_ , whereas within the higher ˙VO _2peak_ category values tend to be underestimated.

The few studies comparing end consumer smartwatches found similar yet slightly greater error rates. Previous researchers investigated the ˙VO _2peak_ provided by the Garmin Forerunner 920XZ (a preceding model of the smartwatch employed here) and observed a MAPE of 7.3% in individuals with a mean ˙VO _2peak_ of 50.3±8.1 ml∙min ^−1^ ∙kg ^−1^ using similar testing procedures as in our study 10 . Klepin and colleagues also applied similar testing procedures and found the MAPE for a smartwatch model by Fitbit (Fitbit Charge 2, Fitbit Inc., San Francisco, CA, USA) was 9.1% with a mean ˙VO _2peak_ of 47.6 ml∙min ^−1^ ∙kg ^−1^ 11 . Our experimental procedures differ from previously performed studies as we include reliability testing of the criterion measure as well. The reliability analysis allows us to compare the error that practitioners should expect when measuring runners twice with the criterion measure (e. g., pre- or post a training period) and when using the smartwatch.

In the given sample, for a runner with a ˙VO _2peak_ of 50 ml∙min ^−1^ ∙kg ^−1^ , the percent variability of the criterion measure is 3.5%, corresponding to an absolute variability of 1.75 ml∙min ^−1^ ∙kg ^−1^ . For a runner with a ˙VO _2peak_ of 60 ml∙min ^−1^ ∙kg ^−1^ this variability is 4.0% (2.4 ml∙min ^−1^ ∙kg ^−1^ ). When employing the criterion measure, any changes of ˙VO _2peak_ smaller than 1.75 to 2.4 ml∙min ^−1^ ∙kg ^−1^ (depending on the level of ˙VO _2peak_ ) should therefore be interpreted cautiously, at least in the given sample and test set-up. In individuals with a ˙VO _2peak_ of 45 to 55 ml∙min ^−1^ ∙kg ^−1^ , variability of the smartwatch and the criterion measure are similar (at least in the given sample) and can be employed interchangeably to assess ˙VO _2peak_ . In individuals with a ˙VO _2peak_ > 55 ml∙min ^−1^ ∙kg ^−1^ or<45 ml∙min ^−1^ ∙kg ^−1^ , the criterion measure shows lower variability than the smartwatch and can therefore better detect smaller changes in ˙VO _2peak_ .

### Usefulness and limitations of V̇O _2peak_ measurement for training in runners

Changes in ˙VO _2peak_ allows runners to evaluate the effectiveness of their previous training procedures with regards to maximal oxygen consumption, however in this case the validity of the provided ˙VO _2peak_ values need to be considered for assessing meaningful changes in ˙˙VO _2peak_ .

For example, when using the smartwatch derived ˙VO _2peak_ measurement, (and based on the present data) a runner with a ˙VO _2peak_ of 50 ml∙min ^−1^ ∙kg ^−1^ will need a change of at least 2 ml∙min ^−1^ ∙kg ^−1^ to be confident that the displayed change may represent a “true” physiological change and not a measurement error due to low validity. Based on our data, runners with a greater baseline ˙VO _2peak_ (>60 ml∙min ^−1^ ∙kg ^−1^ ) will need a change in ˙VO _2peak_ of at least 3.5 ml∙min ^−1^ ∙kg ^−1^ . When using the present smartwatch model, any smaller change should be interpreted with caution when evaluating the response of ˙VO _2peak_ to training.

Based on the miniature design and advanced technology, the smartwatch allows more frequent assessment of ˙VO _2peak_ than it would be possible with laboratory measurement such as stationary or portable gas analysis. Among other factors 25 26 regular (bio-)feedback 27 (e. g., concerning ˙VO _2peak_ changes) may ensure a certain level of adherence to training procedures for some runners.

˙VO _2peak_ often also serves as an anchor measurement to prescribe exercise intensity 7 . For example, exercise at an intensity of 40–60% of ˙VO _2peak_ is considered as “moderate,” whereas an intensity of 60–80% of ˙VO _2peak_ is considered as “vigorous (hard)” according to the American College of Sports Medicine guidelines for exercise testing and prescription 28 . However large variation in homeostatic perturbations (e. g., oxygen uptake kinetics, blood lactate responses) have been reported across multiple studies for exercise performed within those percentages of ˙VO _2peak_ 7 . Consequently, applying fixed percentages of ˙VO _2peak_ to define exercise intensity have shortcomings for normalizing between individuals owing to large inter-individual variation in response 7 . Future studies need to further elaborate the individual response to exercise prescribed as fixed percentages of ˙VO _2peak_ or whether individual percentages of ˙VO _2peak_ are more beneficial to prescribe training procedures.

In summary, while ˙VO _2peak_ measurements obtained by a smartwatch might reveal changes in training adaptation (acknowledging that favorable adaptations such as peak cardiac output or mitochondrial oxidative capacity can occur without improvements in ˙VO _2peak_ 6 ), using ˙VO _2peak_ as an anchor measurement to prescribe exercise intensity has limited applicability in guiding training procedures owing to large inter-individual variations in response.

### Limitations

We investigated healthy and comparably fit individuals with a ˙VO _2peak_ ranging from 38–61 ml∙min ^−1^ ∙kg ^−1^ and did not include participants with higher or lower cardiorespiratory fitness. Cautious interpretation is warranted when transferring our results to other populations, e. g., cardiac patients with altered heart dimension and/or function or individuals with exceptional cardiac dimensions such as elite athletes. Also, our set-up was designed for runners and not for cycling or other sports; therefore we advise to test the validity of ˙VO _2peak_ measurements in different sports involving different movement patterns than running. Additionally, future studies might evaluate whether more running sessions alter the validity of the provided V̇O _2peak_ measurements. Furthermore, future studies should also evaluate if the validity is affected by performing runs of different duration or intensity and in different weather and environmental conditions (e. g., frequent strong headwind or running on sand). The reason for less valid ˙VO _2peak_ estimations of the smartwatch>55 ml∙min ^−1^ ∙kg ^−1^ or<45 ml∙min ^−1^ ∙kg ^−1^ are currently elusive and need further investigation.

As our aim was to test the validity of a smartwatch to estimate ˙VO _2peak_ for end consumer purposes, we did not enter each runner’s peak heart rate into the smartwatch software since estimations with formulas are subject to error 29 and recreational runners often do not know their actual peak heart rate. Therefore, the present results might be different when entering a runner’s true peak heart rate into the software. Additionally, the results may also differ when runners wear a heart rate belt that may assess the heart rate more accurately than the optical heart rate monitor, especially at higher running velocity.

## Conclusions

In the given group of runners as well as the applied testing procedures and within the ˙VO _2peak_ range of 45 and 55 ml∙min ^−1^ ∙kg ^−1^ , the mean absolute percentage error when validating against the criterion measure is 4.1%. The criterion measure revealed a coefficient of variation of 3.5% in this range of ˙VO _2peak_ .

˙VO _2peak_ measurement with the smartwatch in runners with lower (<45 ml∙min ^−1^ ∙kg ^−1^ ) or higher (>55 ml∙min ^−1^ ∙kg ^−1^ ) ˙VO _2peak_ should be judged cautiously due to higher error rates between the smartwatch and the criterion measure.
